# Value of Neuroanesthesiology Fellowship Training

**DOI:** 10.7759/cureus.10943

**Published:** 2020-10-14

**Authors:** Alfred C Ma, Javed Siddiqi, David Ninan

**Affiliations:** 1 Founder and President, Mansfield International College, Fullerton, USA; 2 Anesthesiology, Riverside University Health System Medical Center, Moreno Valley, USA; 3 Neurological Surgery, Riverside University Health System Medical Center, Moreno Valley, USA

**Keywords:** clinical neuroscience, graduate medical education, academic anesthesiology

## Abstract

Rapid technology evolution has led to new challenges for the anesthesiologist in neurosurgical practice. This trend resulted in training in neuroanesthesiology to adapt to the changes. Neuroanesthesiology fellowship training has increasingly received the auspicious attention of graduates from anesthesia residency programs. Competency in neurological surgical procedures requires a multidisciplinary approach with anesthesiologists that hold profound knowledge in neurological sciences.

## Introduction and background

Enhanced learning experiences in neuroanesthesiology and neuro critical care are a part of the changing sphere of clinical neurosciences training [1]. Adaptation to technological advancements and complexity of disease treatment requires anesthesia practitioners to acquire specialty-specific skills to participate in the team approach to care. Furthermore, this observation concurs with the Accreditation Council for Graduate Medical Education’s (ACGME) development of neuroanesthesiology milestones for fellowship training accreditation. ACGME has established seven accredited anesthesiology subspecialties fellowship training programs namely, adult cardiothoracic anesthesiology, clinical informatics, critical care medicine, regional anesthesiology and acute pain medicine, obstetric anesthesiology, pain medicine (multidisciplinary), and pediatric anesthesiology. And expectantly, the neuroanesthesiology milestone is soon to come.

Neurosurgical procedures are extremely delicate and require a collaborative effort to ensure safety and success. Technologically fueled interventional neuroradiology is particularly challenging for anesthesiologists to practice in new settings and demandingly complex procedures [2]. The development of functional and minimally invasive procedures has also accelerated the demand for anesthesiology in the neurosurgical sphere. Such increasing emphasis and expectations for optimal operative conditions prompt anesthesiologists to increase their skills and knowledge to expert levels.

Therefore, multidisciplinary collaboration between neurosurgery and anesthesia during training has become critical for anesthesia practitioners. Furthermore, the success and strength of any neuroanesthesiology program can only be enhanced through collaboration among faculty from the departments of neurosurgery, neurology, anesthesiology, and critical care medicine [3]. Such collaboration is not limited to residency training, as it extends into subspecialty fellowship training to create clinical and research anesthesiology leaders concentrated in the neurosciences and critical care fields.

## Review

Capacity enhancement continues to be a trend in graduate medical education at teaching hospitals. After clinical residency training, anesthesiologists encounter dilemma of whether a subspecialty fellowship is necessary to strengthen her skill sets and competitiveness in the job market. Pursuing a fellowship is yet another training commitment after lengthy residency training. The debate continues if a year or even three more years of subspecialty fellowship training is necessary in today’s challenging and rapidly advancing medical careers.

How critical is neuroanesthesia fellowship?

Anesthesia for neurological procedures requires an advanced understanding of anatomy and physiology of the central nervous system and the pathophysiology of related diseases that are likely to change in response to other diseases, such as space-occupying lesions, trauma, and infection [4]. Anesthesia for neurological diseases requires particular attention to anesthesia-induced changes, such as hemodynamic related perfusion to the central nervous system. Maintenance of physiological conditions is critical to optimizing surgical conditions and preventing further insult to existing pathology.

Conditions such as vasogenic and cytotoxic cerebral edema require an extensive understanding of pathophysiological reasoning and must be treated accurately and appropriately. The anesthesiologist is required to provide optimal operating conditions and to be capable of early detection and proper management of intraoperative events and complications. As such, any disturbances must be corrected to ensure a smooth operational intervention. Emergence should be well and rapidly controlled to establish assessment immediately after the procedure.

Neurosurgery is meticulous and microscopically complex. Anesthesiologists who specialize in this field should understand the fundamentals of neurosurgical procedures and be familiar with critical aspects of the surgery [5]. Communication between the surgical team and anesthesiologist is critical and requires a full understanding of the specialty context. Surgeons and anesthesiologists ought to engage in these cases through academic discussion and continue their collaboration throughout postoperative patient care. This team approach can only be achieved through ample clinical experience with a variety of neurosurgical procedures during the fellowship training period.

What is the goal of neuroanesthesia fellowship?

The goal of neuroanesthesiology fellowship is to provide advanced technological and clinical training in anesthesia related to neurological diseases, including advanced understanding of neurological related physiology, pharmacology, and pathophysiology [6]. Clinically, trainees will also become familiar with neurosurgical procedures and monitoring techniques. Presently, several programs offer a neuroanesthesiology fellowship to provide extra training in the subspecialty even if not yet accredited.

At the end of the neuroanesthesiology fellowship program, the graduate will become skilled in performing preoperative assessment of patients with neurological and neurosurgical diseases. Graduates will be competent in developing and executing an appropriate anesthetic plan that includes monitoring techniques and provides perioperative patient management. Graduates will participate in the neurosurgical patients’ perioperative care and will function as part of a contemporary multidisciplinary neurocritical care team [7]. Fellows will also acquire the research skills to plan and conduct ethical and systematic research in both clinical and basic sciences for publication. Upon completion, fellows are board-eligible for the United Council of Neurologic Subspecialties Neurocritical Care examination and have accumulated a research- and publication-based foundation for their clinical and academic career.

Fellowship market in anesthesia

The mission of fellowship training is to produce advanced-level competence in clinical subspecialties. The ACGME introduced competency-based milestones for resident and fellowship education necessary for independent practice in some specialties. Thus, fellowship programs are established to provide research and educational experience to enhance trainees’ clinical and teaching skills. During the 2019 academic year, the ACGME tabulated there were 1442 anesthesiology senior residents and 1070 fellows in the training pipeline [8]. Estimates are that over 70% of anesthesia residency trainees embark in one of the seven accredited anesthesiology subspecialties fellowship training programs and many more programs pending accreditation, such as the neuroanesthesiology fellowship [9]. The trend of fellowship after residency is growing and was clearly induced by the need to adapt to technology’s rapid advancement in many specialized surgical disciplines.

Milestone development

The Council on Perioperative Neuroscience Training (ICPNT) stated, it is critical for fellows to master the cognition and skills to utilize various monitors, such as fiberoptic intubations, intracranial pressure monitoring, regional cerebral oxygenation monitoring, placement of jugular bulb catheters, and transcranial doppler ultrasonography [10]. Fellows are required to learn basic interpretation of neuroradiological imaging as well, including CT scans, MRIs, and angiograms of brain and spinal cord for tumors, trauma, and vascular lesions and to understand the principles and techniques of interventional neuroradiology. Interpreting intraoperative evoked potentials monitoring and their intraoperative use during brain and spinal cord surgery is a part of fellowship training, which covers somatosensory evoked potentials (SSEPs), motor evoked potentials (MEPs), visual evoked potentials (VEPs), brainstem auditory evoked potentials (BAEPs) and electroencephalography (EEG) [11]. Experiences in neurointensive care and stroke unit are also part of the fellowship training.

The ACGME commissioned the Society for Neuroscience in Anesthesiology and Critical Care (SNACC) to adapt the ACGME anesthesiology milestones for use in neuroanesthesiology graduate medical education and training milestones. Since then, 12 neuroanesthesiology-specific milestones in five major ACGME domains are recommended. These milestones include medical knowledge, practice-based learning and improvement, and interpersonal and communication skills [12]. The recommended milestones are not limited to resident training, as they also establish a foundation for specialty fellowship training.

In the international sphere, developed countries such as Japan and Canada also have a structure adaptive process in advanced neuroanesthesiology training [10,13]. Although the length of training varies, the syllabi are similar and consistent with the ACGME milestone.

## Conclusions

Over the last two decades, growth in the neuroscience field has been exponential. The care of neurosurgical patients requires specialized neuroanesthesiologist. Advanced and complex neurosurgical procedures require the understanding of disease pathology and procedure-specific knowledge. Anesthetic techniques may influence neuropathological conditions, and anesthesia care for neurological related surgeries mandate a thorough preoperative assessment, as these are often pathologically complicated. Participation and spending extra time in subject-specific neuroanesthesiology training is critical. Fellowship provides confidence and skills required to deal with advancements in technology and increased complexity of care. Such training also grants trainees the credentials for team building and patient care.
